# Synergetic Phase Modulation and N‐Doping of MoS_2_ for Highly Sensitive Flexible NO_2_ Sensors

**DOI:** 10.1002/advs.202410825

**Published:** 2024-12-04

**Authors:** Jiyun Kim, Mengyao Li, Chun‐Ho Lin, Long Hu, Tao Wan, Ayad Saeed, Peiyuan Guan, Zijian Feng, Tushar Kumeria, Jianbo Tang, Dawei Su, Tom Wu, Dewei Chu

**Affiliations:** ^1^ School of Materials Science and Engineering University of New South Wales (UNSW) Sydney NSW 2052 Australia; ^2^ School of Chemical Engineering University of New South Wales (UNSW) Sydney NSW 2052 Australia; ^3^ School of Science The Royal Melbourne Institute of Technology (RMIT) Melbourne VIC 3000 Australia; ^4^ Department of Applied Physics The Hong Kong Polytechnic University Kowloon Hong Kong China

**Keywords:** hydrazine, MoS_2_, N‐doping, NO_2_ sensor, phase modulation

## Abstract

Molybdenum disulfide (MoS_2_) is a promising electronic material owing to its excellent electrochemical features, high carrier mobility at room temperature, and widely tunable electronic properties. Here, through precursor engineering and post‐treatments to tailor their phase and doping, electronic characteristics of MoS_2_ are significantly modified. It is found that 2H semiconductor phase with nitrogen doping (N‐doping) in flexible gas sensors constructed with Ag electrodes exhibits the highest sensitivity of ≈2500% toward 10 ppm of NO_2_. This sensitivity is ≈17‐ and 417‐folds higher than that of 2H MoS_2_ without N‐doping, and mixed phases with metallic 1T and semiconductor 2H phase, respectively. Comprehensive experimental investigations reveal mechanisms underlying this record sensitivity, that is, the use of N‐doped 2H MoS_2_ sensors not only significantly suppresses dark current but also effectively enhances electron transfer to NO_2_ molecules. Moreover, density function theory calculations underpin the experimental results, confirming that N_2_H_4_ molecules from the precursor solution not only promote phase transition but also enable N‐doping during post‐treatments, thus boosting sensing capability. This work, for the first time, reveals the synergistic effect of phase modulation and N‐doping of MoS_2_, which can be readily used in other flexible electronic applications, advancing MoS_2_‐based electronics to a new stage.

## Graphical abstract

In this study, a novel synthetic fabrication of MoS_2_ to control the phase transition and chemical modification by nitrogen atoms stemming from hydrazine molecules is proposed. The resulting MoS_2_ gas sensor shows an excellent sensitivity toward NO_2_ outperforming other recent reports, providing new perspectives on improving the transition‐metal‐dichalcogenides‐based gas sensors and showing great potential for the next‐generation gas sensing applications.

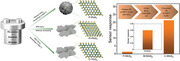

## 1 Introduction

High‐performing and low‐cost gas sensors have been extensively demanded for environmental monitoring, personal safety protection, and industrial manufacturing. Traditional chemiresistive sensors based on semiconducting oxides have outperformed in the commercial market due to excellent sensitivity and long‐term reliability. However, they still encounter unsolved issues such as requiring high temperatures (ranging from 200 to 600 °C) for operation, and relatively complex fabrication. To revolutionize gas sensing devices, it is imperative to comprehensively design gas sensors featuring high performance, wearability, low cost, and large‐scale manufacturability, while addressing the issues found in traditional chemiresistive sensors using semiconducting oxides.

Nanostructured materials with large surface areas and facile processing through printing or spin‐coating are considered one of the most promising candidates to serve as active layers in sensing electronics.^[^ ^1^, ^2^ ^]^ Among various nanostructured materials, the intriguing properties of two‐dimensional (2D) transition metal dichalcogenides (TMDs) molybdenum disulfide (MoS_2_) have recently demonstrated great prospects in versatile applications, including catalysis,^[^ ^3^, ^4^ ^]^ energy storage,^[^ ^5^ ^]^ electronics,^[^ ^6^, ^7^ ^]^ and sensors.^[^ ^8^, ^9^ ^]^ Moreover, MoS_2_ has featured remarkable optical and electrochemical sensing capabilities due to its inherent high surface‐to‐volume ratio, widely tunable electronic structure, and excellent air stability.^[^ ^10^, ^11^ ^]^ Furthermore, owing to its exceptional biosafety, MoS_2_ is regarded as a promising material in fabricating wearable sensing electronics for human bodies.^[^ ^12^, ^13^ ^]^ These compelling features make MoS_2_ stand out among 2D TMDs for the next generation of sensing applications.

To date, the most extensively researched MoS_2_ gas sensors are based on field‐effect transistors (FETs) employing either single or multiple layers of MoS_2_ nanosheets serving as the charge transport channel.^[^ ^6^, ^14^, ^15^ ^]^ However, the complex FETs fabrication process often results in unsatisfied reproducibility and high costs, which still suffer from low sensitivity. In the pursuit of improving sensing properties, methods such as Van der Waals junction,^[^ ^16^ ^]^ heterostructure,^[^ ^17^ ^]^ functionalization,^[^ ^18^ ^]^ morphological modification,^[^ ^19^ ^]^ and chemical doping^[^ ^20^ ^]^ have been developed. In addition, there have been numerous studies on the effect of doping, and a few studies on the effect of phase structuring on gas sensing performance. For example, some phase structure simulation studies^[^ ^21^, ^22^ ^]^ using either 1T^[^ ^23^ ^]^ or mixed 1T/2H phase^[^ ^15^ ^]^ proposed that 1T phase may facilitate faster electron transfer and favorable interaction with gas molecules. However, as the 1T phase is thermodynamically unstable due to its relatively high energy configuration, a robust approach to synthesizing a sustainable 1T phase at room temperature remains yet to be resolved. The difficulty in controlling the MoS_2_ phase also makes it challenging to verify a clear structure‐function relationship in gas sensing. Moreover, the correlation between sensing performance, phase structures (such as metallic 1T and semiconducting 2H), and impurity doping, remains unclear due to the absence of a study that incorporates both techniques.

Herein, we devise a facile synthetic strategy to prepare three types of MoS_2_ with different phases and doping, offering a platform to systematically investigate their electronic characteristics and corresponding gas sensing performance. It was found that pure 2H semiconductor phase without N‐doping (pristine MoS_2_) and 1T/2H mixed phase (mixed MoS_2_) exhibits low gas sensing capability toward NO_2_ due to inadequate charge transfer pathways and extremely high dark current respectively, while the sensors based on fully converted semiconducting 2H phase with N‐doping from mixed phase (converted 2H MoS_2_) features ≈17 and 417 times higher sensitivity (≈2500%) than pristine and mixed‐phase MoS_2_ devices, surpassing the up‐to‐date reported MoS_2_‐based NO_2_ sensors by ≈10 times. Through thorough mechanism investigations, we found that the synergetic effect of phase modulation and N‐doping from N_2_H_4_ molecules on MoS_2_ can significantly alter the electronic states, thereby enhancing charge transfer efficiency to NO_2_ molecules in the gas sensing process. Furthermore, given the potential of inkjet printing technology for miniaturizing wearable electronic devices and achieving large‐scale production yield, we successfully demonstrated printed Ag electrodes for gas sensors with excellent sensing performance, which satisfies the requirements for the next generation of NO_2_ gas sensors. Therefore, this work not only achieved a formulation for a MoS_2_ sensor that exhibits ultra‐high sensitivity toward NO_2_, but also importantly revealed the synergistic effect of phase modulation and N‐doping of MoS_2_, which can be readily used in other flexible electronic applications, advancing MoS_2_‐based electronics to a new stage.

## 2 Results and Discussion

**Figure 1:**
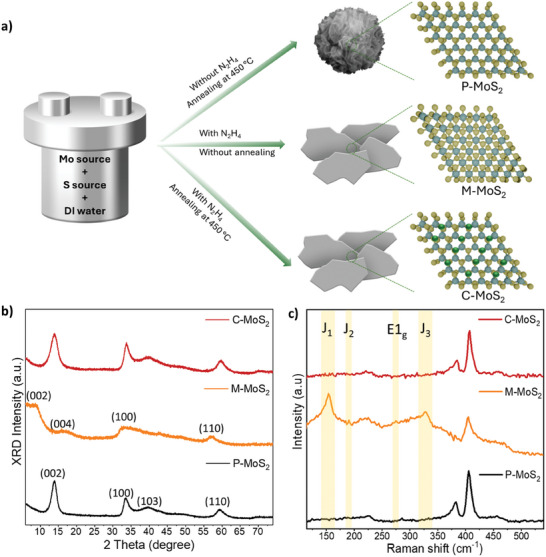
a) Schematic illustration of N_2_H_4_‐assisted doping strategy during hydrothermal synthesis procedure. b) XRD patterns and c) Raman spectrum of all three MoS_2_ showing crystal structure.

In this study, three types of MoS_2_ were synthesized by controlling precursors and post‐treatments, named pristine, mixed phase (1T/2H), and converted 2H MoS_2_ (detailed in the Experimental Section and Figure S1, Supporting Information). As shown in **Figure** **1**a, pristine 2H MoS_2_ was prepared by mixing molybdenum and sulfur sources in deionized (DI) water without other additives followed by hydrothermal synthesis and annealing post‐treatment at 450 °C in a tube furnace (labeled as P‐MoS_2_). For the synthesis of the mixed 1T/2H phase, beyond the molybdenum source and sulfur source, N_2_H_4_·H_2_O was dropped into mixtures and deionized (DI) water (labeled as M‐MoS_2_). Here, N_2_H_4_ plays two roles in tailoring the electronic properties of MoS_2_. First, N_2_H_4_ is a powerful reducing agent that donates a pair of electrons to electron‐deficient species due to strong nucleophilicity, which induces 1T/2H mixed phase, impeding a sudden or gradual phase transformation at ambient condition due to its thermodynamical stability.^[^ ^24^ ^]^ Second, after further annealing post‐treatment, intercalated N_2_H_4_ could affect the electronic states and increase its susceptibility to NO_2_ gas. It is noted that mixed 1T/2H phase MoS_2_ was not subjected to annealing post‐treatment. To produce pure 2H phase MoS_2_ with N‐doping, the mixed‐phase MoS_2_ with N_2_H_4_ intercalation was annealed at 450 °C for 1 h under H_2_/Ar gas for phase conversion and N‐doping from N_2_H_4_ decomposition (labeled as C‐MoS_2_).

To verify the crystal structure of different types of MoS_2_ powders, X‐ray diffraction (XRD) was conducted as shown in Figure 1b. P‐MoS_2_ demonstrates two main diffraction peaks located at the angles of 13.8°, 33.5°, and 39.8° corresponding to (002), (100), and (103) planes, respectively, which is indicative of its hexagonal structure (2H) phase.^[^ ^25^ ^]^ After intercalating with N_2_H_4_, the (002) peak of M‐MoS_2_ shifted to a lower angle of 9.2°, suggesting that lattice expansion is driven by intercalation of N_2_H_4_. Furthermore, an additional peak corresponding to 1T (004) plane appeared at 17°,^[^ ^26^ ^]^ which confirms that the mixed 1T/2H phase has been successfully achieved. C‐MoS_2_ exhibits an XRD pattern characteristic of the 2H phase, with the (002) and (100) peaks slightly shifted to higher angles by 0.1° and 0.2°, respectively, compared to P‐MoS_2_ (Figure S2, Supporting Information). This shift is attributed to the annealing process used for phase conversion, which may cause lattice strain modification induced by sulfur vacancies and the incorporation of smaller nitrogen atoms (≈65 pm) as dopants.^[^ ^27^ ^]^ Raman spectroscopy was further carried out for all samples to confirm structural phases (Figure 1c). It is observed that P‐MoS_2_ exhibits significant peaks at the wavenumbers of 382 and 407 cm^−1^, corresponding to the in‐plane E^1^_2_ _g_ vibration mode and the out‐of‐plane A_1__g_ vibration mode of MoS_2_, which indicates Mo─S phonon vibration of semiconducting phase MoS_2_. Contrarily, M‐MoS_2_ reveals additional peaks at the wavenumbers of 150, 205, 278, and 325 cm^−1^, representing Mo─S phonon modes of 1T phase corresponding to J_1_, J_2_, E_1g_, and J_3_. For C‐MoS_2_, the absence of characteristic 1T phase vibration modes confirms that mixed phase was fully converted into the semiconductor 2H phase. Notably, E^1^_2g_ and A_1g_ vibration modes of C‐MoS_2_ are upshifted (Figure S3, Supporting Information) due to compressive strain caused by N‐doping in the lattice, which is consistent with the XRD result showing N‐doping (Figure S2, Supporting Information).

**Figure 2:**
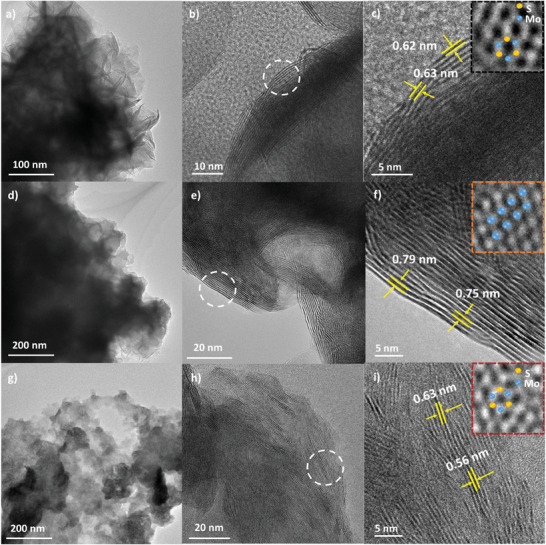
TEM images of a–c) P‐MoS_2_, d–f) M‐MoS_2_, and g–i) C‐MoS_2_ with lattice spacing associated with (002) planes. Insets of (c), (f), and (i) are large magnification of TEM image to observe atomic structure, indicating that P‐MoS_2_ and C‐MoS_2_ have honeycomb structure while M‐MoS_2_ shows octahedral coordination.

Transmission electron microscopy (TEM) was further carried out to confirm the atomic structures of P‐MoS_2_, M‐MoS_2_, and C‐MoS_2_ at the nanoscale. In **Figure** **2**a–c, P‐MoS_2_ exhibits three‐dimensional nanoflower‐like structures assembled by lamellar nanosheets with the average interlayer *d*‐spacing from 0.62 to 0.63 nm, which is in good agreement with previously reported 2H phase MoS_2_.^[^ ^28^, ^29^ ^]^ In addition, the atomic arrangement of P‐MoS_2_ was identified as a honeycomb hexagonal atomic structure (inset of Figure 2c), which is consistent with the XRD result. Figure 2d–f is the TEM images of M‐MoS_2_ showing that layers of nanosheets are uniformly stacked. The morphology has been changed from nanoflowers to nanosheets by adding hydrazine monohydrate, which is attributed to the byproduct of NH_3_ gas induced from N_2_H_4_ that hinders nanosheets from being densified as nanoflower‐like structures.^[^ ^30^ ^]^ Interestingly, the average interlayer d‐spacing of M‐MoS_2_ was slightly enlarged ranging from 0.75 to 0.79 nm compared to P‐MoS_2_ 2H counterpart (≈0.62 nm), which is ascribed to intercalation of N_2_H_4_ species resulting in a slight expansion in interlayers.^[^ ^24^, ^31^ ^]^ Moreover, octahedral coordination was observed in M‐MoS_2_ (Inset of Figure 2f), which confirms the existence of 1T phase in M‐MoS_2_. In Figure 2g–i, C‐MoS_2_ displayed no significant morphological changes following the phase conversion for C‐MoS_2_, which retains nanosheet structures (Figure S4, Supporting Information) and 2H phase (inset of Figure 2i). It was observed that the *d*‐spacing of certain interlayers in C‐MoS_2_ was slightly reduced to ≈0.56 nm, and energy‐dispersive X‐ray (EDX) analysis further verified a uniform distribution of N atoms within the C‐MoS_2_, as shown in Figure S5 (Supporting Information). Based on concurrent analysis, we can conclude that N atoms were successfully doped into the C‐MoS_2_, leading to a reduced interlayer spacing.

**Figure 3:**
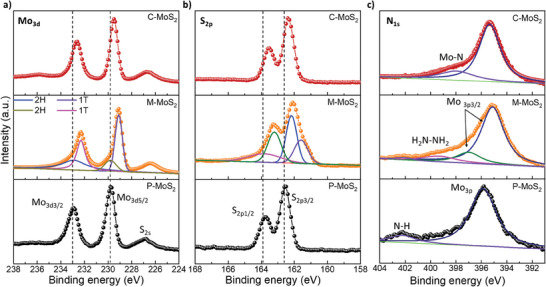
XPS spectrum of P‐MoS_2_, M‐MoS_2_, and C‐MoS_2_ associated with a) Mo3d, b) S2p, and c) N1s, respectively, showing chemical and electronic states.

To further investigate the chemical state and atomic level interaction of all three types of MoS_2_, X‐ray photoelectron spectroscopy (XPS) measurements were carried out. In **Figure** **3**a, the XPS spectra of all three MoS_2_ in the Mo 3d region reveal the binding energies are located at 232.9, 229.6, and 226.7 eV corresponding to Mo^4+^ 3d_3/2_, Mo^4+^ 3d_5/2_, and S 2s, respectively. For M‐MoS_2_, additional binding energies at 232.1 and 299.2 eV associated with 1T phase are observed.^[^ ^24^, ^32^ ^]^ Analogously, as shown in the XPS S 2p spectra in Figure 3b, the binding energies of 163.7 and 162.5 eV corresponding to S 2p_1/2_ and S 2p_3/2_ are detected for P‐MoS_2_ and C‐MoS_2_ while two additional peaks at 163.3 and 161.5 eV were observed for M‐MoS_2_, which implies the existence of 1T phase in M‐MoS_2_. Interestingly, after the phase transition to C‐MoS_2_, the binding energies corresponding to Mo 3d and S 2p orbitals are downshifted by 0.3 eV. In general, lower binding energy implies a higher density of electrons around the core level leading to weakening bond strength with the nucleus, which was evidenced by previously reported works.^[^ ^17^, ^30^ ^]^ Therefore, it is concluded that electrons are effectively transferred to MoS_2_ with the assistance of N_2_H_4_ in the converted pure 2H system. For the N 1s spectrum of all three MoS_2_ (Figure 3c), P‐MoS_2_ shows a distinct peak at 402.2 eV which corresponds to N‐H bonding stemming from residual from Mo and S sources during the synthesis process while it is absent in M‐MoS_2_ and C‐MoS_2_. In the M‐MoS_2_ sample, the peak at 399.3 eV is assigned to the binding energies of N associated with N_2_H_4_.^[^ ^33^ ^]^ After N_2_H_4_ intercalated M‐MoS_2_ was converted into C‐MoS_2_, it can be observed a characteristic peak at 397.95 eV, which is associated with the binding energy of Mo‐N bonding,^[^ ^34^ ^]^ thus confirming N‐doping after phase conversion.

**Figure 4:**
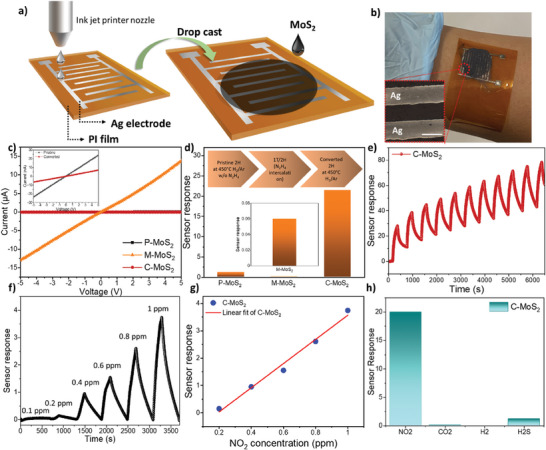
NO_2_ sensor fabrication and sensing performance of all MoS_2_ sensors. a) Schematic illustration of sensor fabrication with inkjet printing method and drop casting of MoS_2_ solution. b) Photo of fabricated sensors that can be flexible and wearable with an inset showing the spacing of interdigitated Ag electrode with a scale bar of 100 µm. c) *I*–*V* characteristics of P‐MoS_2_, M‐MoS_2_, and C‐MoS_2_ deposited on inkjet printed Ag interdigitated electrode under a bias from −5 to 5 V. d) Bar graph representing sensor responses of P‐MoS_2_, M‐MoS_2_, and C‐MoS_2_ sensors under 10 ppm of NO_2_ at RT. e) Dynamic sensing response of C‐MoS_2_ under 10 ppm of NO_2_ at room temperature over continuous 10 cycles. f) Sensor response of C‐MoS_2_ as a function of NO_2_ concentration from 0.1 to 1 ppm. g) NO_2_ concentration dependent sensor responses featuring regression fitting lines of C‐MoS_2_. h) Selectivity test of C‐MoS_2_ at 10 ppm of other target gases.

To explore chemiresistive gas sensing behaviors of different types of MoS_2_ samples, sensing devices were fabricated using an ink‐jet printing technique to print interdigitated silver electrodes on polyimide (PI) films with an electrode spacing of 100 µm. MoS_2_ solution was then deposited onto the substrate via layer‐by‐layer drop casting, as illustrated in **Figure** **4**a,b. The average thickness of deposited MoS_2_ film is ≈7 µm, determined by cross‐sectional scanning electron microscope (SEM) analysis (Figure S6, Supporting Information). Before conducting NO_2_ sensing measurements, the I‐V characteristics of P‐MoS_2_, M‐MoS_2_, and C‐MoS_2_ were first tested to verify the contact behavior between Ag and MoS_2_ films since barrier height at the metal/sensing layer interface significantly impacts sensing performance.^[^ ^35^ ^]^ *I*–*V* characteristics of all three MoS_2_ sensors exhibit a linear current response as a function of voltage (Figure 4c), suggesting that low contact barrier height is formed with Ag (φ = 4.2 eV) in all sensors, which facilitates the gas sensing as charge transfer induced by target gas dominantly contribute to the resistance change.^[^ ^36^ ^]^ Notably, the resistivity of M‐MoS_2_ is found to be two orders of magnitude lower than that of the other two MoS_2_, manifesting the conductive metallic nature of the 1T phase. Moreover, the resistivity of C‐MoS_2_ demonstrates fourfold higher than that of P‐MoS_2_ under N_2_ condition, which might be attributed to a decrease in hole concentration caused by electron donation resulting from N doping via N_2_H_4_.^[^ ^37^ ^]^

To further investigate electrochemical sensing properties, NO_2_ sensing measurement for all MoS_2_ sensors was conducted under a carrying gas of N_2_ with a bias of 5 V at room temperature. The sensor response is defined as (*I*_NO2_/*I*_N2_ − 1), where *I*_NO2_ and *I*_N2_ are current under NO_2_ and N_2_, respectively, and sensor responses of all MoS_2_ sensors are recorded. As shown in Figure 4d, the sensor response was depicted based on the average value of continuous current change (Figure S7, Supporting Information) of all MoS_2_. P‐MoS_2_ presents a sensitivity of 1.5 toward 10 ppm of NO_2_ and the sensitivity dramatically reduced to 0.06 for M‐MoS_2_. Surprisingly, the sensitivity of C‐MoS_2_ was significantly enhanced to 25, featuring 17 and 417 times higher than that of P‐MoS_2_ and M‐MoS_2_, respectively. P‐MoS_2_ and C‐MoS_2_ demonstrate positive sensor responses when NO_2_ is introduced, suggesting a p‐type characteristic generating more hole concentration due to the strong electron‐withdrawing behavior of NO_2_ gas. However, M‐MoS_2_ represents the opposite sensing behavior where resistance increases when exposed to NO_2_ since any gas molecules can interfere with carrier movement in the metallic phase. From the sensing measurement, we can confirm sensing behavior of M‐MoS_2_ is dominated by the 1T phase, which shows a deteriorating performance than pure semiconducting 2H phase MoS_2_ (P‐MoS_2_ and C‐MoS_2_). Compared to up‐to‐date MoS_2_‐based NO_2_ sensors in the literature (**Table** **1**),^[^ ^14^, ^15^, ^34^, ^38^, ^39^, ^40^, ^41^, ^42^, ^43^, ^44^, ^45^, ^46^ ^]^ our C‐MoS_2_ sensor demonstrates extremely higher sensitivity toward NO_2_, indicating that the synergistic effect of phase modulation and N doping plays a crucial role in determining the sensing performance, which will be discussed in a later session.

**Table 1:** Comparison of up‐to‐date chemiresistive MoS_2_‐based NO_2_ sensors at room temperature.

Material	Synthesis method	Fabrication method	Conc [ppm]	Response [%]	LOD [ppm]	*T*_res_/*T*_rec_ [s]	Refs.
MoS_2_ nanoflower	Hydrothermal	Drop cast	10	67.4	NA	N/A	[38]
MoS_2_ nanosheets	Hydrothermal	Spray	100	21	NA	40/48	[39]
MoS_2_ thin film	CVD	CVD	100	10	NA	N/A	[14]
MoS_2_ bilayer	CVD	CVD	10	11	<1	678/318	[40]
PbS/MoS_2_ nanocomposite	Hydrothermal	Drop cast	100	23	NA	200/1300	[41]
MoS_2_/porous Si heterojunction	CVD	Chemical etching, sputtering	50	28	1	N/A	[42]
MoS_2_/CNT	CVD	CVD	25	6	0.002	N/A	[43]
MoS_2_ nanoworm	DC magnetron sputter	DC magnetron sputter	100	10	NA	N/A	[44]
MoS_2_ nanosheets	Hydrothermal	Plasma/drop cast	10	225	0.062	55/323	[45]
MoS_2_ nanoflower	Hydrothermal	Atomic substitution	1	230	0.01	146/52	[46]
MoS_2_ nanoflower	Solvothermal	Drop cast	10	28	0.125	22/109	[34]
MoS_2_ monolayer	commercial	Drop cast	2	25	0.025	10/120	[15]
C‐MoS_2_	Hydrothermal	Drop cast	10	2500	0.13	43.1/301.2	This work

The cycling stability test of C‐MoS_2_ sensors was performed with continuous ten on‐off cycles, confirming the great repeatability of C‐MoS_2_ sensors (see Figure 4e). In Figure 4f, a transient sensor response of C‐MoS_2_ at low NO_2_ concentration from 0.1 to 1 ppm was tested and we observed a linear response toward NO_2_ within this low concentration range, suggesting that it is beneficial for precise NO_2_ detection. Considering the detection level, we extracted the theoretical limit of detection (LOD) based on the “standard deviation” method using a plotted linear fitting line from the sensor responses at low NO_2_ concentrations (0.1–1 ppm). The LOD of C‐MoS_2_ is estimated to be 0.13 ppm, which is lower than that of P‐MoS_2_ (≈0.17 ppm). We further examined thickness‐dependent sensing performance of C‐MoS_2_ (Figure S8, Supporting Information), and it is worth noting that reduced thickness deteriorate sensitivity, which could be attributed to nonuniform MoS_2_ film leading to less surface reaction area. Selectivity is one of the key factors for gas sensors. For C‐MoS_2_, we observed an excellent selectivity with ultrahigh sensitivity toward NO_2_ among other target gases, as shown in Figure 4h, representing a great careful detection of NO_2_ gas.

**Figure 5:**
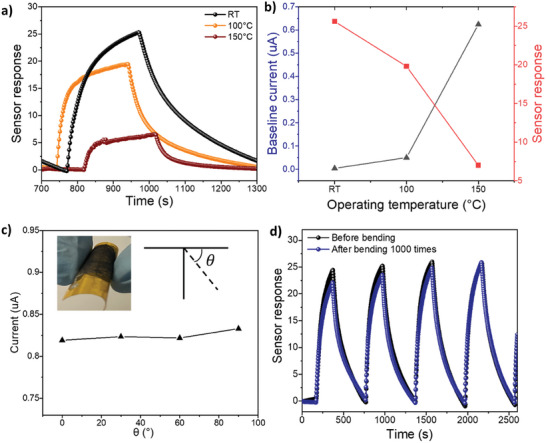
a) Temperature‐dependent dynamic sensing response of C‐MoS_2_ from room temperature (RT) to 150 °C. b) Sensitivity with baseline current of C‐MoS_2_ at different operating temperatures. c) The resistance record of C‐MoS_2_ with respect to different bending degree. d) Dynamic sensing response of C‐MoS_2_ before bending test and after 1000 times bending cycles.

We further explored the use of C‐MoS_2_ sensors in high‐temperature conditions as well as examined the bending properties and durability of our flexible C‐MoS_2_ sensor. In **Figure** **5**a, it is observed that the sensitivity gradually decreases as temperature increases to 150 °C. The sensitivity of C‐MoS_2_ at 150 °C is measured to be 5.5, which is still higher than that of P‐MoS_2_ and other reported MoS_2_ NO_2_ sensors at room temperature (Table 1), indicating a great sensing capability even at high temperatures. It is worth noting that the baseline current increases as the temperature rises, showing a semiconducting characteristic for C‐MoS_2_ (Figure 5b).^[^ ^38^ ^]^ The sensor response gradually reduces as temperature increases since excessive thermal energy accelerates the desorption of NO_2_. Encouraged by the advantages of wearable sensors, we tested the flexibility properties of C‐MoS_2_ by mechanical bending test. The resistance of C‐MoS_2_ showed negligible change at different bending angles up to 90°, as shown in Figure 5c. Furthermore, in the durability test, the performance of the C‐MoS_2_ sensor was maintained even after 1000 bending cycles at 90° (Figure 5d). These results indicate that the C‐MoS_2_ sensor has intact sensing performance at different bent angles during measurement and demonstrates excellent durability under repeated bending.

**Figure 6:**
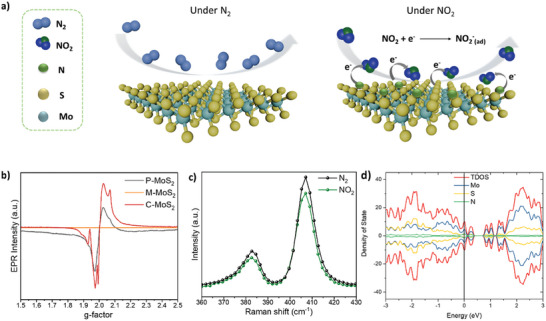
a) Illustration of NO_2_ adsorption mechanism on C‐MoS_2_ surface. b) EPR measurement of all three MoS_2_, representing different paramagnetic behavior. c) In situ Raman spectroscopy measurement of C‐MoS_2_ under N_2_ and NO_2_, respectively. d) Density of states of N coordinated MoS_2_ (C‐MoS_2_) showing the Fermi level located at the valence band in NO_2_ adsorption system.

Intrigued by the superior sensing performance of C‐MoS_2_, we elucidated the sensing mechanism associated with NO_2_ interaction. Chemiresistive sensors are operated by the variance of resistance induced by charge transfer between analytes and the sensing layer, therefore, active sites favoring NO_2_ adsorption and effective charge transfer are key factors in strengthening sensing capability. As depicted in **Figure** **6**a, NO_2_ adsorption can trigger electron transfer from MoS_2_ surface to NO_2_ molecules forming NO_2_^−^ at room temperature, consequently, it substantially lowers resistance in p‐type semiconducting MoS_2_ (P‐MoS_2_ and C‐MoS_2_). In general, large surface areas create favorable conditions generating active sites. However, this work evaluated that the shape transformation from nanoflower (P‐MoS_2_) to stacked nanosheets (M‐MoS_2_ and C‐MoS_2_) reduces the effective surface area exposed to NO_2_. It is evidenced by Brunauer–Emmett–Teller (BET) nitrogen adsorption‐desorption analysis (Figure S9, Supporting Information), which verifies the surface areas of M‐MoS_2_ and C‐MoS_2_ dramatically decreased to 10.3 and 13.3 m^2^ g^−1^, respectively, compared to that of P‐MoS_2_ (39.3 m^2^ g^−1^). Hence, we highlight the importance of efficient charge transport pathways over the effective surface area, which is in fact a critical aspect of improving sensing capability. In previous studies, it is proven that either rich sulfur vacancy or oxidation of Mo^4+^ engineering plays a pivotal role in influencing NO_2_ sensing capability as dangling bonds from unpaired Mo^4+^ or electron‐rich environment driven by MoO_x_ can provide abundant active sites.^[^ ^47^, ^48^ ^]^ In our study, the oxidation state of Mo^6+^ or MoO_x_ in C‐MoS_2_ was not observed in Raman and XPS results above, suggesting that the oxidation of Mo is unlikely to act as active sites for NO_2_ gas; instead, N‐doping is more likely responsible for the enhanced sensing property of C‐MoS_2_. According to the literature report, N dopants can act as new active sites that not only create favorable surface interaction between N‐doped MoS_2_ and NO_2_ gas molecules but also facilitate higher charge transfer, allowing more electron transfer from MoS_2_ to NO_2_. To further verify the effect of magnetic attraction and electron transfer driven by N‐doping, we performed electron paramagnetic resonance (EPR) measurements, as shown in Figure 6b. In the EPR spectra of all three MoS_2_, P‐MoS_2_ presents a g‐factor at 2.001 originating from sulfur vacancies generated during the annealing process.^[^ ^49^, ^50^ ^]^ Interestingly, it is found that M‐MoS_2_ has no signal at the same g‐factor, suggesting the absence of sulfur vacancy defects. In contrast, C‐MoS_2_ demonstrates higher EPR signal intensity than P‐MoS_2_, which can be attributed to excessive unpaired electrons induced by N‐doping.^[^ ^51^ ^]^ Since we confirmed that N‐ doping donates electrons to the C‐MoS_2_ system, charge transfer driven by electron doping is effectively introduced to NO_2_ in C‐MoS_2_, thus significantly enhancing sensor response. As a proof of concept, it is further corroborated by Raman spectroscopy for C‐MoS_2_ to confirm real‐time adsorption of NO_2_ on the active sites of MoS_2_. We examined the Raman spectrum of P‐MoS_2_ as a reference (see Figure S10, Supporting Information) and the Raman spectra of C‐MoS_2_ when exposed to N_2_ and NO_2_, respectively, as shown in Figure 6c. Unlike P‐MoS_2_, the intensity change of both in‐plane vibration (E^1^_2g_) and out‐of‐plane (A^1^_g_) are prominently decreased in C‐MoS_2_ when exposed to NO_2_, which is ascribed to effective electron transfer, reducing electron‐phonon interaction as NO_2_ captures electrons on the surface.^[^ ^52^ ^]^

Density functional theory (DFT) was carried out by using Born–Oppenheimer Molecular Dynamics (BOMD) via the CP2K package to corroborate the interaction and effective charge transfer between NO_2_ and C‐MoS_2_. As shown in Figure S11 (Supporting Information), the NO_2_ molecules were adsorbed on the top of N coordinated with Mo in the C‐MoS_2_ system and charge transfer from C‐MoS_2_ to NO_2_ was verified. As a comparison with the S‐vacancy defective MoS_2_ model (P‐MoS_2_), both Fermi level of P‐MoS_2_ and C‐MoS_2_ is located within the valence band under exposure of NO_2_, confirming that both materials are p‐type semiconductor with holes as the majority carriers (Figure 6d and Figure S12, Supporting Information).^[^ ^53^ ^]^ However, the Fermi level of N atoms coordinated MoS_2_ (C‐MoS_2_) in the NO_2_ environment is found to shift further toward the valence band compared to P‐MoS_2_, indicating that the N coordination results in a higher generation of holes that lead to a superior p‐type transport. As a result, C‐MoS_2_ features an exceptional sensing response outperforming up‐to‐date MoS_2_ sensors, thus showing great promise for phase modulation and N‐doping synergetic effect strategy.

## 3 Conclusion

In summary, we developed a novel procedure to fabricate MoS_2_ with controllable electronic properties through phase modulation and heteroatom doping, and then flexible sensors with excellent sensing performance are successfully achieved. Through comprehensive analysis of chemical information and electronic states of pristine, mixed phase (1T/2H), and converted 2H MoS_2_ with N‐doping, we concluded that pure 2H phase MoS_2_ without N‐doping and mixed phase MoS_2_ weakens sensing capability due to poor charge transfer toward NO_2_ and elevated baseline current density driven by metallic 1T phase, respectively. However, converted 2H MoS_2_ with N‐doping surprisingly demonstrated the highest sensitivity (≈2500%) toward 10 ppm of NO_2_ at room temperature. Through systematic mechanism investigations, we, for the first time, reveal that the synergistic effect of phase modulation and N‐doping play a crucial role in achieving high sensitivity, which can be readily extended to other TMDs for wearable sensors.

## 4 Experimental Section

### Materials Preparation

All the chemicals were purchased from Sigma‐Aldrich. Pristine and hydrazine assisted MoS_2_ powder were synthesized by a hydrothermal synthesis approach. 0.53 g of ammonium heptamolybdate [(NH_4_)_6_Mo_7_O_24_·H_2_O] and 0.46 g of TAA (C_2_H_5_NS) were mixed into beaker as Mo and S source, respectively. For hydrazine treated MoS_2_ powder, 0.15 mL of hydrazine monohydrate (N_2_H_4_·H_2_O) was added in the beaker to make the molar ratio N_2_H_4_/Mo of 1:1, followed by adding DI water to make the total volume of 40 mL. The mixture was continuously stirred for 1 h and then the precursors were transferred to clean 50 mL stainless‐steel autoclaves to heat up at 200 °C for 48 h. The final product was cooled down to room temperature and the suspension was taken out and washed with mixture of DI water and ethanol five times, followed by drying in a vacuum oven at 50 °C overnight to obtain the black loose powder.

### Preparation of Inkjet‐Printed Ag Interdigitated Electrode

The conductive Ag printing ink was purchased from Sigma‐Aldrich and printing of Ag electrode was carried out using an inkjet printer (Fujifilm Dimatix DMP‐2800). The cartridge was designed to dispense droplets with a volume of 10 pL. The commercial Ag ink was printed onto polyimide (PI) substrate with 100 µm gap of the electrode, followed by further annealing in an oven at 300 °C for 1 h.

### Fabrication of MoS_2_ Embedded Gas Sensor

As‐synthesized MoS_2_ powder was suspended in ethanol and dispersed with a sonication bath for 2 h. To further break down the Van der Waals force between MoS_2_ layers, the solution was placed in a probe sonicator with high power for 1 h to disperse the powder uniformly. The final suspension was deposited by drop casting onto the printed Ag electrode, followed by annealing at 90 °C overnight.

### Evaluation of MoS_2_ NO_2_ Sensing Properties

The sensor measurements were performed as follows. For any gas sensing measurements, pure nitrogen gas was used as a background gas. The gas flow rate was controlled by mass flow controllers (Bronkhorst) but kept the total gas flow rate at 500 sccm. The analyte gases (CO_2_, in N_2_, H_2_, in N_2_, H_2_S, 20 ppm in N_2_) were purged into the gas chamber (Linkam) with a flow rate of 500 sccm. For the gas sensing measurements, two Au probes were separately placed on top of the two arms of inkjet‐printed Ag interdigitated electrode with an applied constant potential of 5 V. The dynamic current curve of MoS_2_ sensor was recorded by Keithley 4200A‐SCS. For temperature dependent sensing measurement, the sensor was placed onto the stage of Linkam where high temperature was varied by a temperature controller. The devices were taken out for the gas sensing measurements normally for about 1–2 h each time, and then stored in a dry desiccator for the rest of the time.

### Characterizations and Measurements

The surface morphology of all samples was examined by JEOL JSM‐IT500 field‐emission SEM system. Crystal structure and element distribution were analyzed with TEM and EDS measurements. The specific surface area was obtained by Nova Touch LX2 BET surface area analyzer. All the samples were degassed under vacuum at 250 °C for 8 h prior to the N_2_ adsorption/desorption experiment at a temperature of 77 K. For crystal structural analysis, XRD was carried out using a Bruker D8 ADVANCE diffractometer with Cu Ka (lambda = 0.15406 nm) radiation. Surface chemistry was analyzed by FTIR Spectrometer (PerkinElmer) and XPS and EPR spectra of all MoS_2_ powder were obtained at 120 K with a frequency of 9.85 × 109 Hz.

### Computational Details

Vienna Ab initio Simulation Package (VASP) based on the generalized gradient approximation (GGA) with the Perdew–Burke–Ernzerhof (PBE) function as the exchange‐correlation energy function. (The projector augmented wave potentials with the cutoff energy of 450 eV was used. The conjugate gradient scheme optimizes the atom coordinates until the force is less than 0.01 eV Å^−1^.) For the mono‐layer MoS_2_ (unit cell *a* = 12.3709 Å, *b* = 11.0173 Å, *c* = 14 Å) relaxation calculations, the 2 × 2 × 1 of k‐points were given for Monkhorst–Pack BZ calculations to achieve energy convergence. Meanwhile, the D3 correction was applied for all the layer structure calculations to consider the Van der Waals effect.

## Conflict of Interest

The authors declare no conflict of interest.

## Supporting information



Supporting Information

## Data Availability

The data that support the findings of this study are available from the corresponding author upon reasonable request.
