# Supplementary material for: Synergetic Phase Modulation and N‐Doping of MoS2 for Highly Sensitive Flexible NO2 Sensors
Source: Adv Sci (Weinh). 2024 Dec 4;12(4):2410825. doi: 10.1002/advs.202410825 (PMC11775570; doi:10.1002/advs.202410825)
Supplement: Supplementary file 1 — Supporting Information [file ADVS-12-2410825-s001.docx]

Supporting Information

**Synergetic Phase Modulation and N-Doping of MoS_2_ for Highly Sensitive Flexible NO_2_ Sensors**

*Jiyun Kim, Mengyao Li, Chun-Ho Lin*, Long Hu*, Tao Wan*, Ayad Saeed, Peiyuan Guan, Zijian Feng, Tushar Kumeria, Jianbo Tang, Dawei Su, Tom Wu*, Dewei Chu*

**
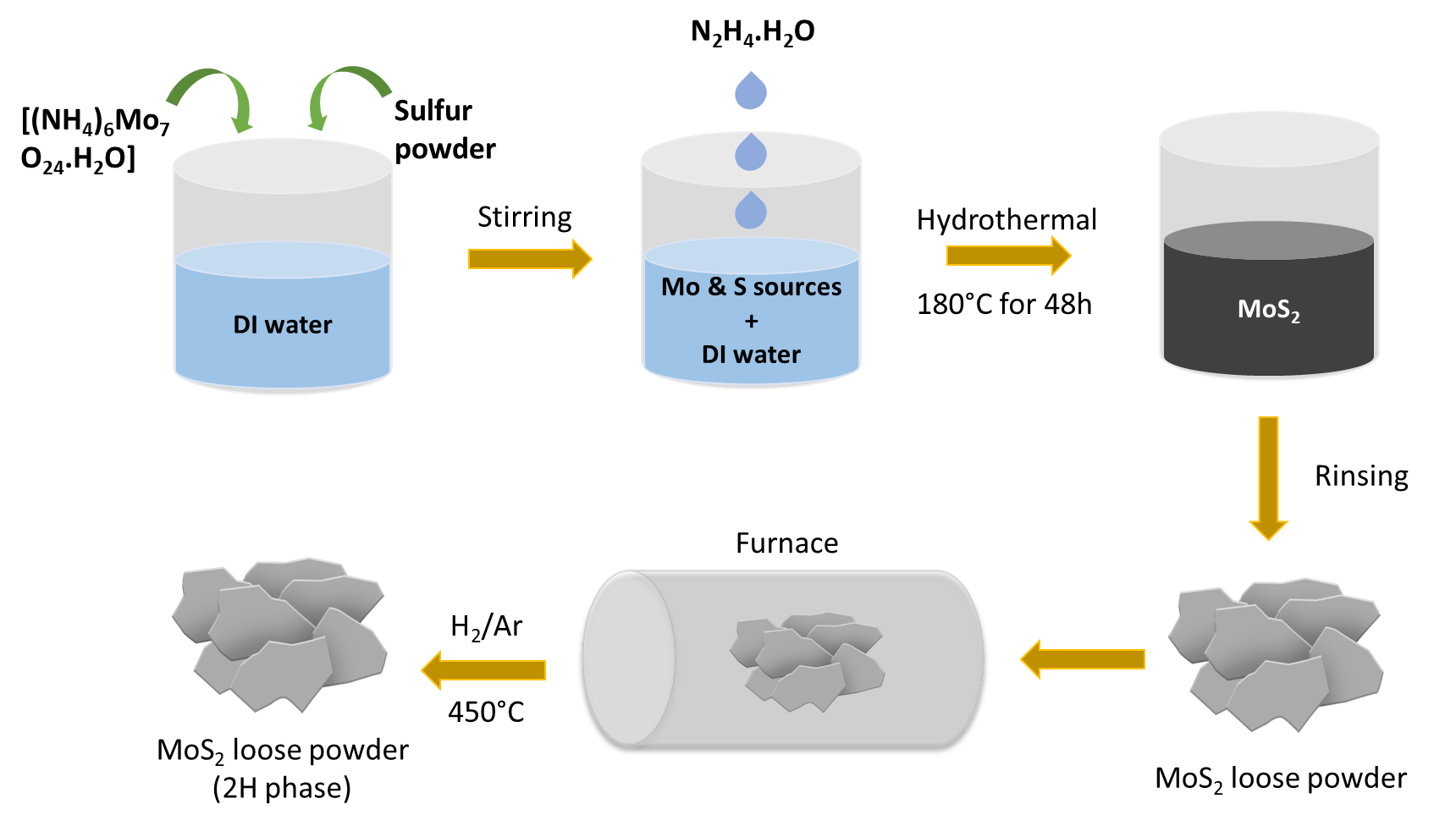
**

Figure S1. Illustration of hydrothermal synthesis procedures of hydrazine treated MoS_2_


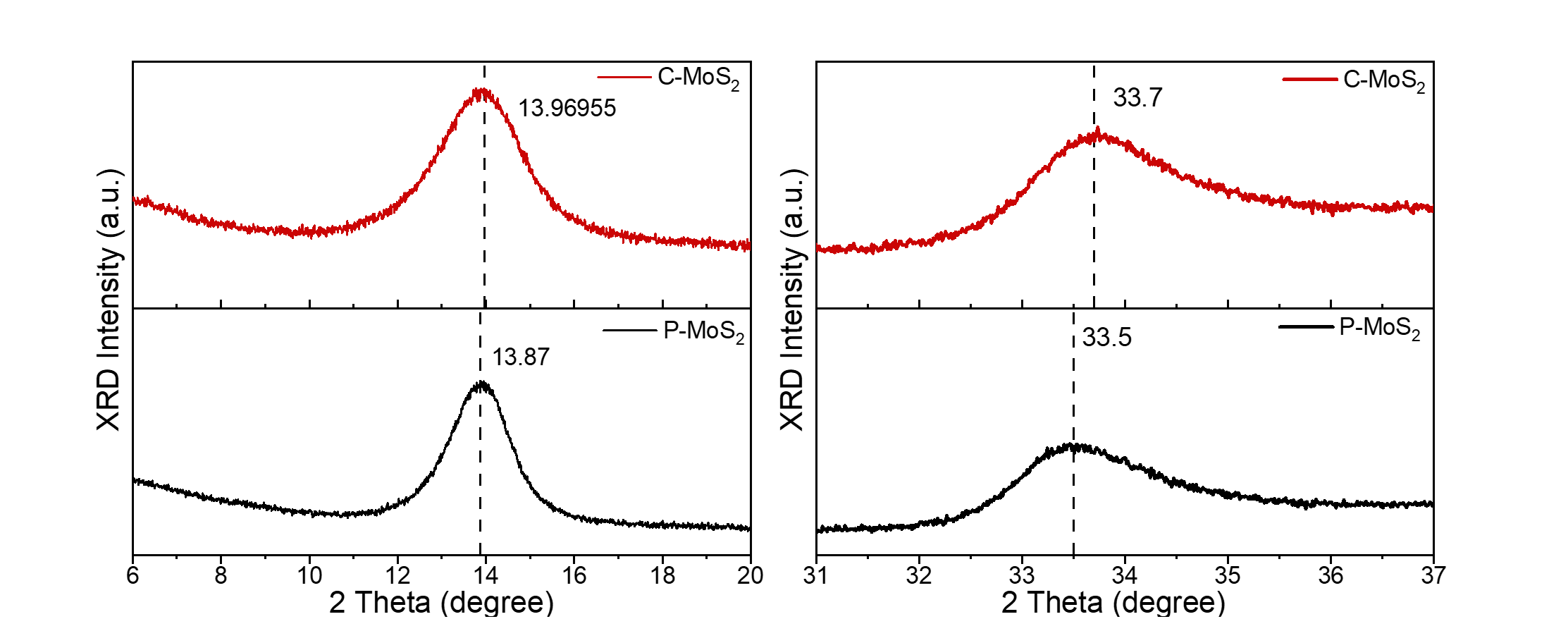


Figure S2. Enlarged XRD peaks corresponding to (100) and (002) planes in P-MoS_2_ and C-MoS_2_, respectively


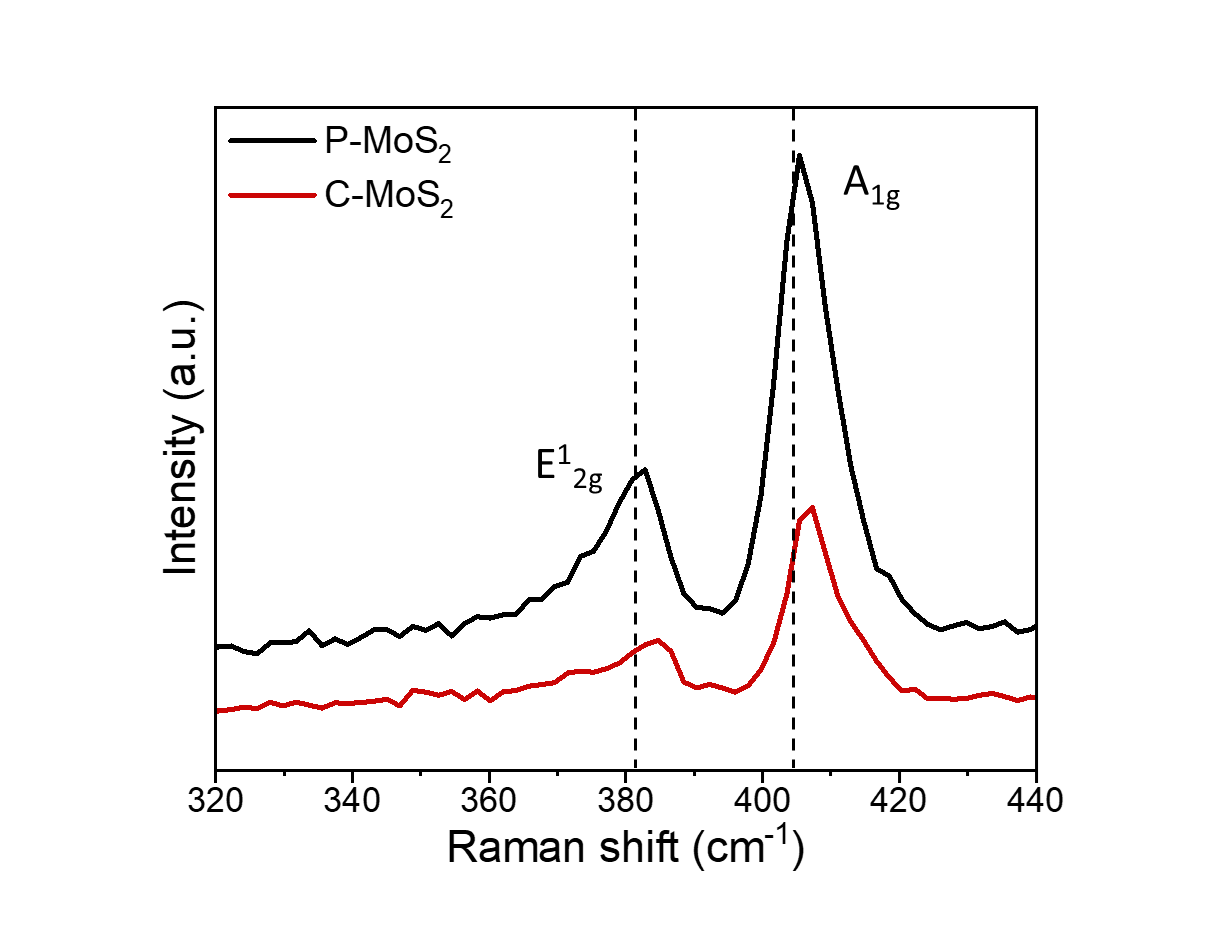


Figure S3. Enlarged E^1^_2g_ and A_1g_ vibration modes of P-MoS_2_ and C-MoS_2_.


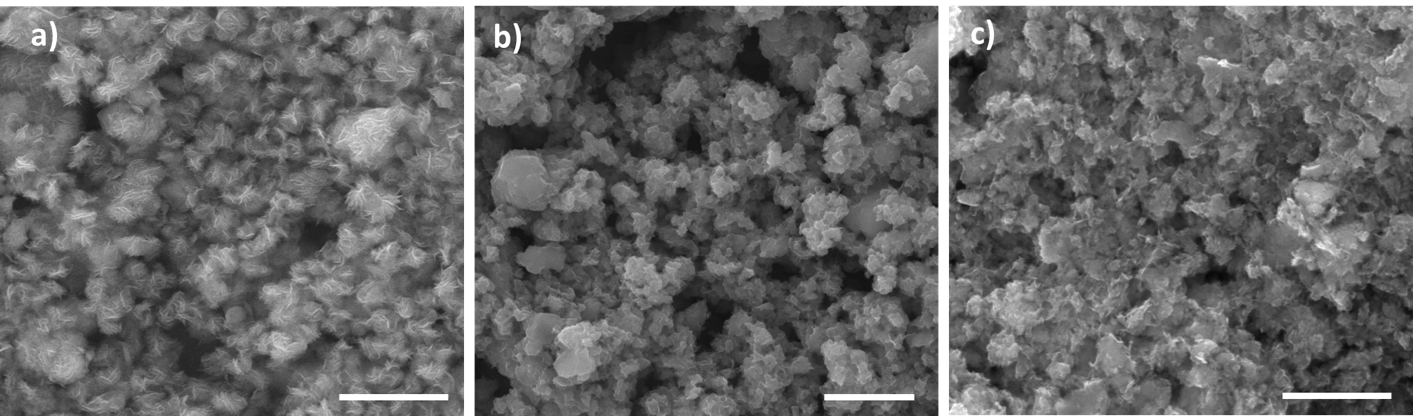


Figure S4. Top-view SEM images of a) P-MoS_2_, b) M-MoS_2_, and c) C-MoS_2_ with a scale bar of 1µm.


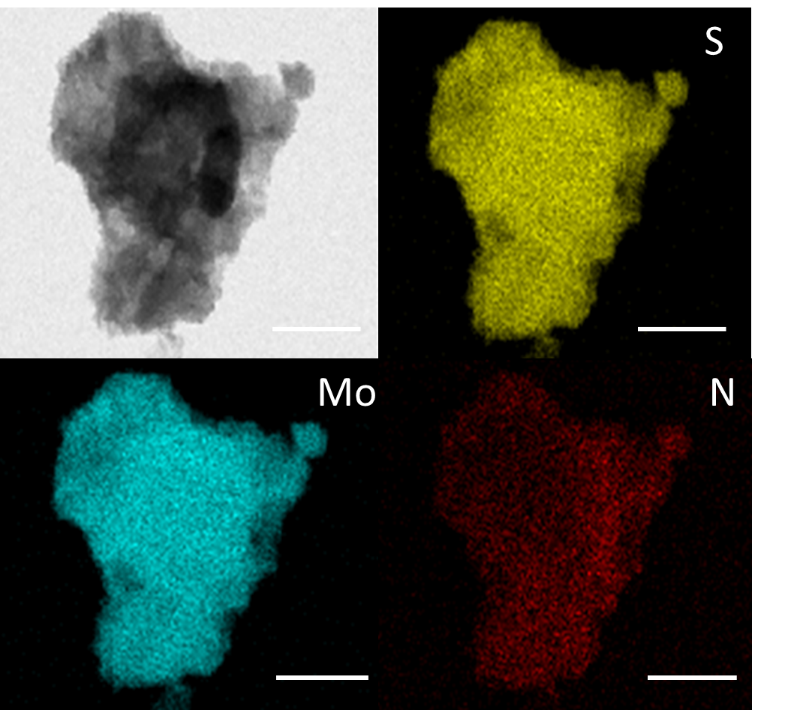


Figure S5. EDX analysis of C-MoS_2_, confirming that Mo, S, and N atoms are well distributed with a scale bar of 250 nm.


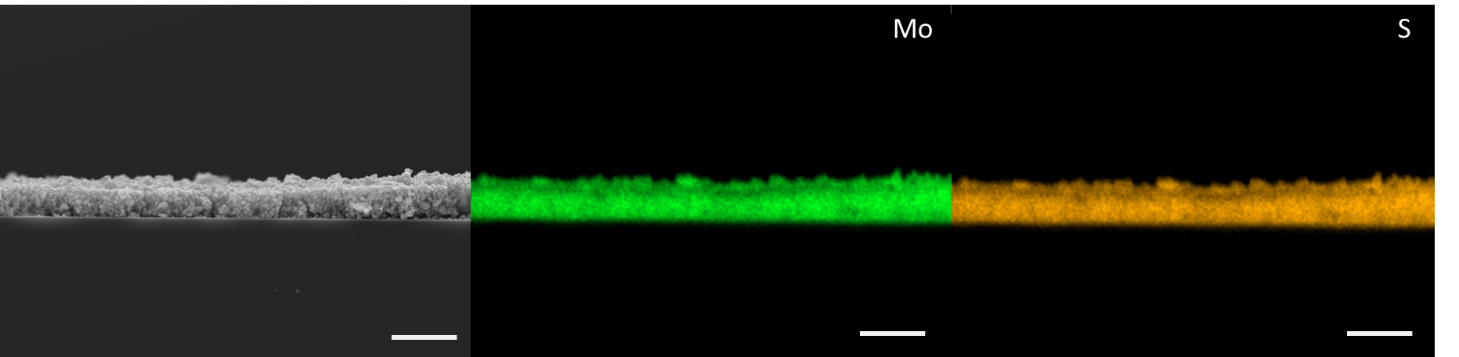


Figure S6. Cross-sectional SEM image and EDX analysis of the C-MoS_2_ film with scale bars of 10 µm.


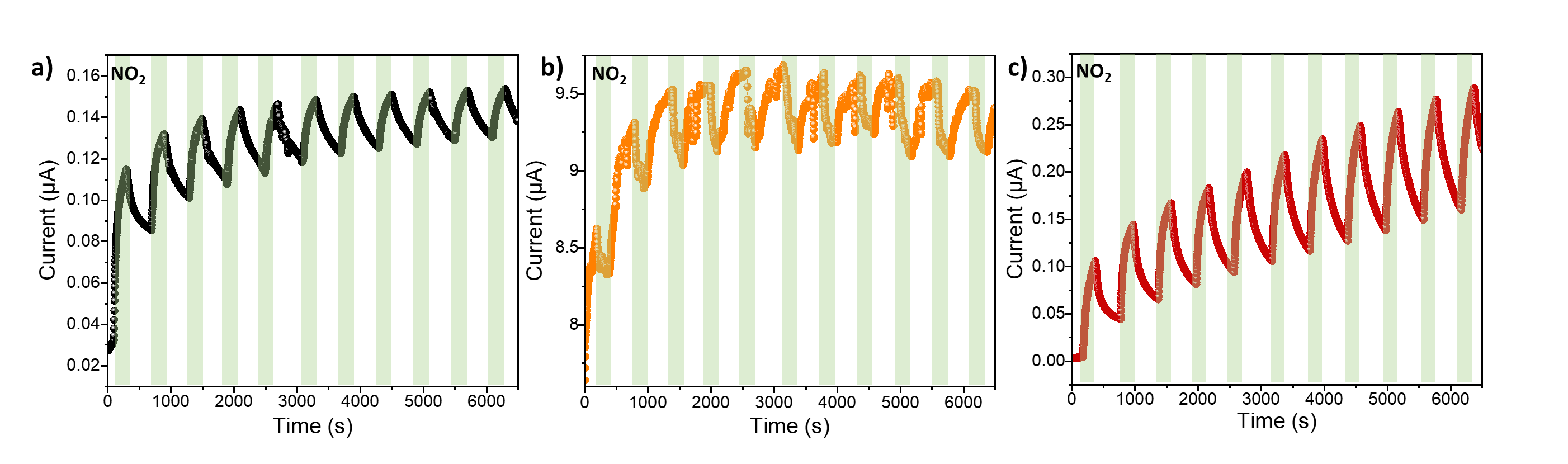


Figure S7. Dynamic current curve of P-MoS_2_, M-MoS_2_, and C-MoS_2_ sensor at 10 ppm of NO_2_ with a bias of 5 V under N_2_.

Figure S8. Sensor response of C-MoS_2_ with 1, 5 (default in this manuscript), and 10 layers deposited via drop casting.


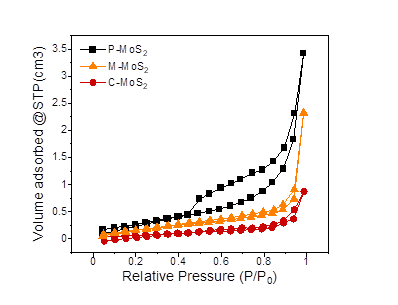


Figure S9. BET nitrogen adsorption isotherms of P-MoS_2_, M-MoS_2_, and C-MoS_2_ representing the surface area.


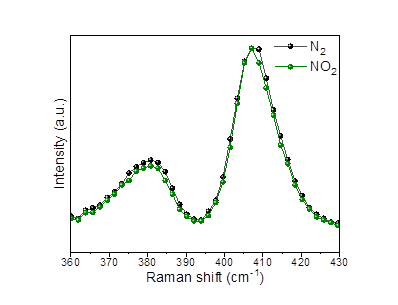


Figure S10. Real-time Raman spectroscopy measurement of P-MoS_2_ under N_2_ and NO_2_, respectively.


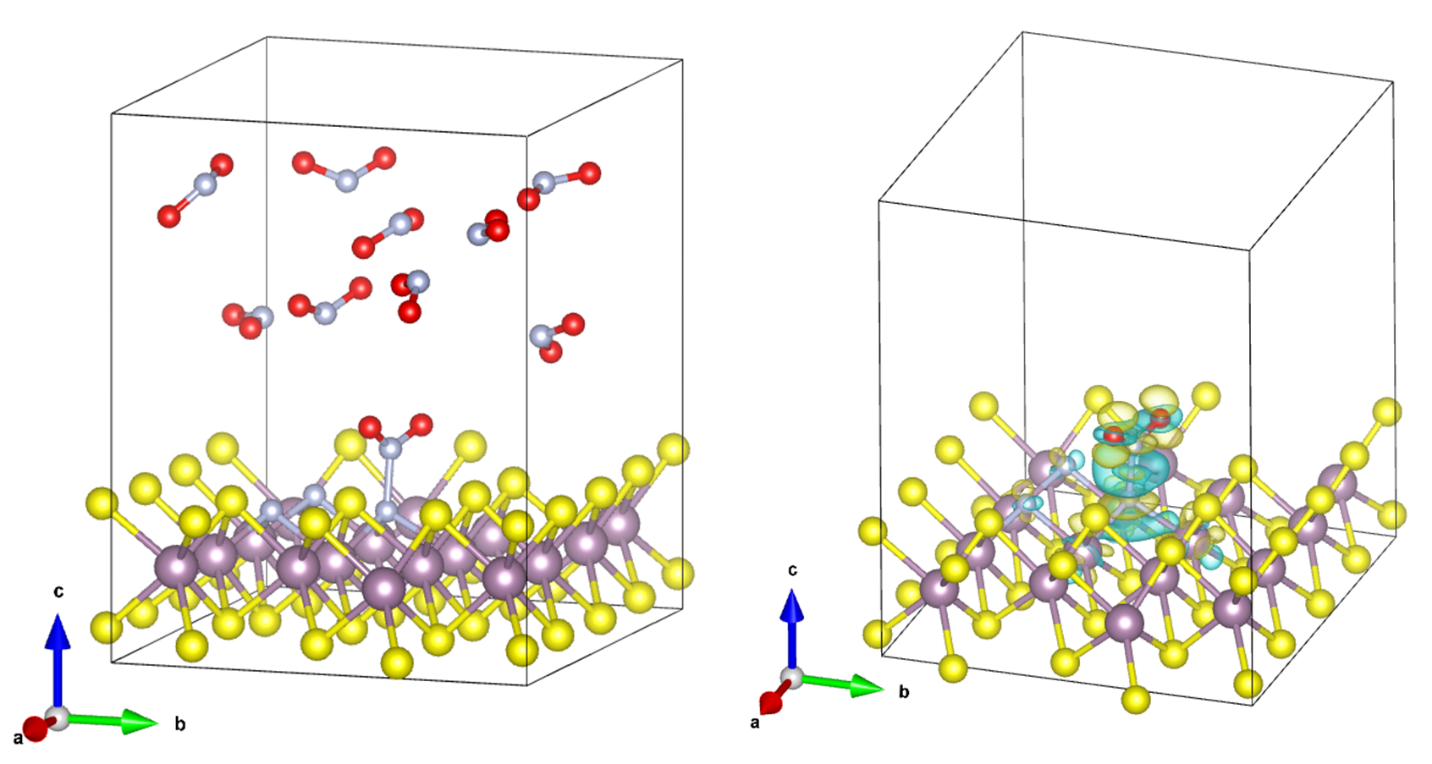


b)

a)

Figure S11. a) Initial model of 10 NO_2_ molecules on the top of monolayer 3 N atoms coordinated MoS_2_. After 10 ns BOMS calculation, the static frame of the 10 NO_2_ molecules on the top of monolayer 3 N atoms coordinated MoS_2_ (C-MoS_2_). b) Electron density difference plot between 3 N atoms coordinated MoS_2_ (C-MoS_2_) to NO_2_ (Electron density isosurface = 0.003|e|/Bohr^3^).


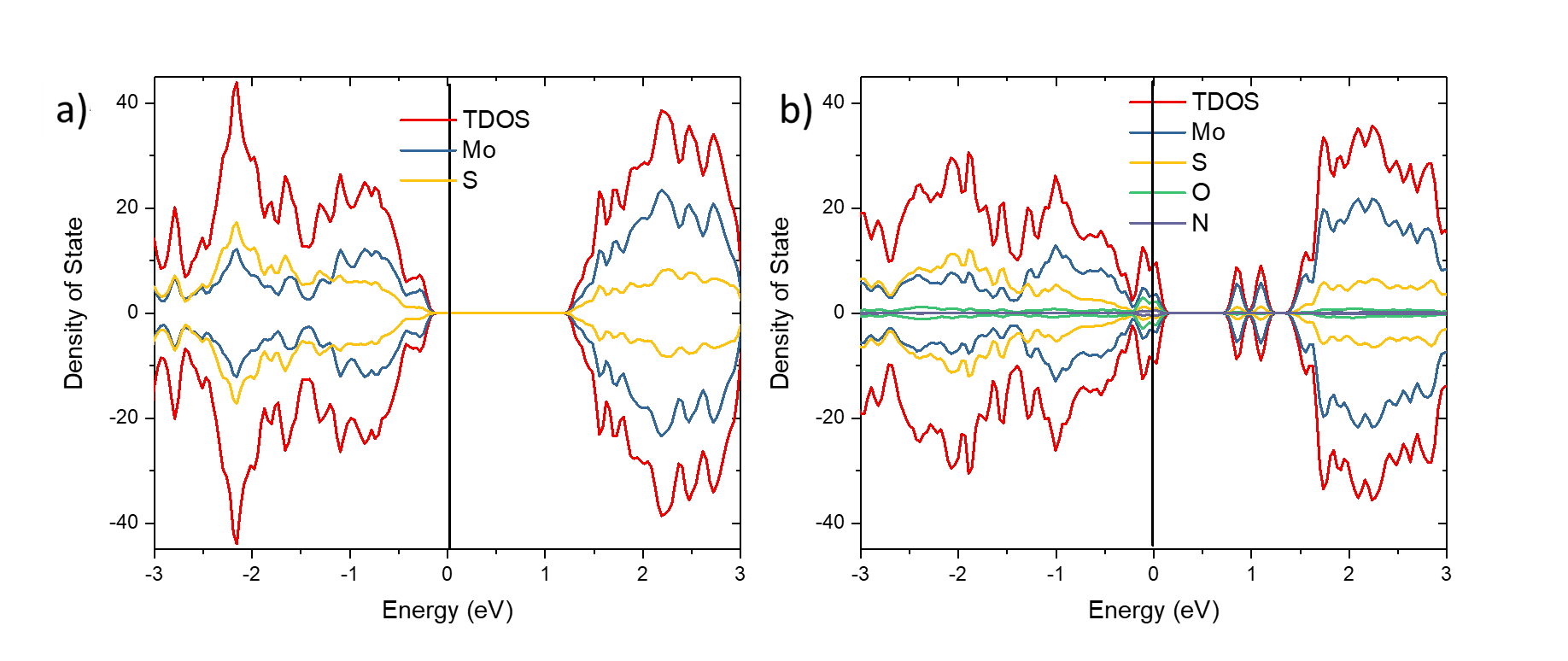


Figure S12. Density of States (DOS) of (a) defect free MoS_2_, (b) sulfur vacancy defective MoS_2_ (P-MoS_2_). The black solid vertical line at 0 eV is the Fermi level.
